# A meta-analysis to observe silage microbiome differentiated by the use of inoculant and type of raw material

**DOI:** 10.3389/fmicb.2023.1063333

**Published:** 2023-02-24

**Authors:** Roni Ridwan, Mohammed Abdelbagi, Ahmad Sofyan, Rusli Fidriyanto, Wulansih D. Astuti, Ainissya Fitri, Mohammad M. Sholikin, Rohmatussolihat, Ki A. Sarwono, Anuraga Jayanegara, Yantyati Widyastuti

**Affiliations:** ^1^Research Center for Applied Zoology, National Research and Innovation Agency (BRIN), Cibinong, Indonesia; ^2^Animal Feed and Nutrition Modelling (AFENUE) Research Group, Department of Nutrition and Feed Technology, Faculty of Animal Science, IPB University, Bogor, Indonesia; ^3^Department of Animal Nutrition, Faculty of Animal Production University of Khartoum, Khartoum North, Sudan; ^4^Study Program of Nutrition and Feed Science, Graduate School of IPB University, Bogor, Indonesia; ^5^Research Center for Animal Husbandry, National Research and Innovation Agency (BRIN), Cibinong, Indonesia; ^6^Department of Nutrition and Feed Technology, IPB University, Bogor, Indonesia

**Keywords:** silage, meta-analysis, microbiome, bacterial diversity, lactic acid bacteria

## Abstract

Silage fermentation is naturally carried out by lactic acid bacteria (LAB) to mainly produce lactic acid (LA) and other organic acids as preservatives. Along with fermentation time, the growth of LAB will replace and suppress undesirable microorganisms. This meta-analysis study aimed to explore silage microbiome differentiated by LAB inoculants and type of raw materials. A total of 37 articles with 185 studies and 475 datasets were used for building up the meta-database. Data were subjected to the mixed model methodology. The parameters observed were silage quality and silage microbiome post-ensiling process. Results revealed that four bacterial genera along with *Weissella* dominated the post-ensiling process. The addition of lactic acid inoculants in the silage has increased the abundance of *Lactobacillus* spp. and decreased the Shannon index significantly. Moreover, the abundance of both *L. plantarum* and *L. buchneri* increased, and subsequently, *Weissella, Pseudomonas, Proteobacteria*, pH value, ammoniacal nitrogen (NH_3_-N), coliforms, and the yeasts were decreased significantly due to the addition of LAB inoculants in silage (*p* < 0.05). Environmental factors such as temperature affected the existence of *Pseudomonas, Exiguobacterium*, and *Acinetobacter*. However, the dry matter, LA, acetic acid (AA), the ratio of LA to AA, and the LAB population were enhanced significantly (*p* < 0.05). Among the LAB types, the lowest abundance of *Pseudomonas* was due to the LAB group, while the lowest abundance of *Weissella* and *Proteobacteria* was due to the addition of the combined LAB group. In conclusion, the addition of LAB is effectively enhancing the silage microbiome and silage quality by altering bacterial diversity and the metabolic products of the silage materials for safe preservation.

## 1. Introduction

Silage represents one of the promising feed products for ruminants in the world. The practice of making silage started a long time ago; however, ensiling remains the main method for forage preservation to provide a palatable and available feed source that is less affected by various weather conditions (Zhang et al., 2021). Moreover, silage is used to seek efficiency in milk production on farms using silage in the diet of livestock throughout the year (Bernardes and Rêgo, 2014). Efforts on providing good quality feed all year round, such as silage, is necessary to guarantee the good welfare of farm animals (Keeling et al., 2019).

Ensiling is a complex process involving the role of microorganisms, which can be referred to as the microbiome, that largely determine the quality of silage produced. Starter cultures such as silage inoculum are important additives to ensure the perfect ensiling process. It is believed that lactic acid bacterial (LAB) strains are good candidates for advancing the fermentation process of their fast growth rate, high resistance to low pH conditions, and quick production of desirable substrates such as lactic acid (LA) over a wide range of temperature changes. Inoculation of low-temperature tolerant LAB at ensiling could stimulate favorable fermentation and reconstruct the bacterial community for better preservation of highly moist oat silage nutrients (Chen et al., 2020). Jaipolsaen et al. (2022) reported the importance of identifying suitable starter cultures, understanding the natural flora of epiphytic LAB on plants, and applying them together to optimize cost-effective silage production. Among the most used starter cultures of LAB is *Lactobacillus plantarum* (Keshri et al., 2018; Mu et al., 2020). Completely different microbial flora and their successions during ensiling were observed using a recent methodology that provides information regarding the microbial processes underlying silage formation to achieve high-quality silage production (Guo et al., 2018).

Microorganisms in silage are important indicators of silage quality; however, their presence is strongly influenced by the available metabolite derived from the type of material used for the manufacture of silage. There are various kinds of forage materials, with various metabolite contents, used to make silage. Inoculation of different starter cultures altered the microbial composition and fermentative metabolites in ensiled whole-crop corn silage in very different ways. The correlations between metabolites and bacterial species can provide important scientific information on screening targeted LAB for the modulation of silage fermentation. Profiling of silage microbiome and metabolome can improve our current understanding of the biological process of silage formation (Xu et al., 2019) and can be used to evaluate ensiled forages not only in terms of fermentation quality but also based on nutritional and functional metabolites that are beneficial to animal health and welfare (Guo et al., 2018). We hypothesized that several LAB inoculants could have various effects on the silage microbiome and silage quality based on the raw materials used in silage making. Therefore, the present meta-analysis study aimed to explore silage microbiome differentiated by LAB inoculants and types of raw material used in silage making.

## 2. Materials and methods

### 2.1. Structuring the database

In this study, the database was built by collecting the datasets from previously published articles. In detail, literature was obtained through a number of steps, i.e., identification and screening, and then valid articles were inserted into an excel spreadsheet. During the identification process, the search engines, namely Google, Scopus, and Google Scholar, were used for searching the datasets of the previously published articles. The keywords used were lactic acid bacteria, silage quality, bacterial diversity, and fermentation.

The identification process was carried out based on the titles of the collected articles. In this stage, we put general criteria in the article that would be involved in the database. These criteria are as follows: (1) Article must be written in the English language; (2) Only published articles; (3) Collected article must contain a control treatment with at least one experiment of LAB addition among their treatments; and (4) The collected articles must contain at least one parameter on silage microbiome or at least one parameter on silage quality. Here, in this stage, we obtained 181 articles.

The process was continued by scanning the entire abstract of each of the collected articles to ensure that the article is valid to be used in this stage. At this stage, 54 articles were obtained. The screening process was done by reading carefully the entire content of each collected article to determine which one of the collected articles is valid to be inserted into the database. The literature obtained at this stage was 37 articles with 185 studies and 485 datasets. All valid articles were inserted into an excel spreadsheet. Information about articles used in the database is presented in Table 1.

**Table 1:** Studies used in the meta-analysis database.

**References**	**Study**	**Experiments**	**Substrates**	**Dose (Log CFU/ g FM)**	**Type of LAB**	**Ensiling Period (d)**	**Replicates**
Mu et al. (2022)	1	8	Mixture	0–6	*L. plantarum, L. buchneri*, and combination	45	3
Chen et al. (2021)	2	8	Stylo and rice straw	0–5	*L. plantarum*	30	3
Xiong et al. (2022)	3	2	Oat	0–6	*L. plantarum, L. rhamnosus*, and *L. paracasei*	60	3
Mu et al. (2020)	4	8	Mixture	0–5	*L. farciminis*	60	3
Chen et al. (2020)	5	5	Mixture	0–5	*L. plantarum*	30	5
Du et al. (2021)	6	4	Mixture	0–5	*L. plantarum*	60	5
Xu et al. (2020)	7	2	Mixture	0–6	*L. plantarum*	90	3
Wang et al. (2016)	8	1	Rice straw	0–9	Combination	90	3
Oskoueian et al. (2021)	9	7	Rice straw	0–6	*L. plantarum, L. farciminis, L. salivarius*, and *L. reuteri*.	30	3
Guan et al. (2020a)	10	4	Napier grass	0–6	*L. buchneri* and *L. rhamnosus*	60	3
Guo et al. (2020)	11	2	Bur clover and annual rye grass	0–6	Combination	60	3
Li P. et al. (2018)	12	6	Mixture	0–6	*L. plantarum*	30	3
Wang et al. (2016)	13	5	Bur clover and annual rye grass	0–6	Combination	60	3
Jaipolsaen et al. (2022)	14	9	Napier grass, corn, and Mixture	0–14	*L. plantarum*	42	3
Romero et al. (2018)	15	8	Corn	0–5	Combination	100	3
Romero et al. (2017)	16	4	Oat	0–6	Combination	217	3
You et al. (2022)	17	1	Alfalfa	0–5	*L. plantarum*	34	9
Benjamim da Silva et al. (2021)	18	2	Corn	0–11	Combination	99	3
Ogunade et al. (2017)	19	2	Corn	0–5	Combination	128	4
Li et al. (2021)	20	5	Rice straw	0–11	Combination	60	3
Du et al. (2022)	21	14	Paper Mulberry	0	-	60	3
Keshri et al. (2018)	22	15	Corn	0–6	*L. plantarum*	60	3
Ni et al. (2017)	23	3	Soybeans	0–6	Combination	60	3
Guan et al. (2021)	24	6	Corn	0–6	*L. plantarum*	60	3
Bai et al. (2021)	25	5	Alfalfa	0–5	*L. plantarum*	60	4
Zhang (2018)	26	5	Alfalfa	0–6	*L. plantarum*	60	3
Zi et al. (2021)	27	6	King grass	0–5	*L. plantarum*	60	3
Wang et al. (2019)	28	6	*Neolamarckia cadamba*	0–9	*L. plantarum*	60	3
Zhao et al. (2021a)	29	4	King grass	0–5	*L. plantarum*	60	3
Wang et al. (2019)	30	1	Moringa leaves	0–4.5	*L. plantarum*	30	3
Li D. X. et al. (2018)	31	4	Alfalfa	0–6	*L. plantarum*	60	3
Santos et al. (2015)	32	3	Corn	0–6	*L. plantarum*	90	3
Huo et al. (2021)	33	1	Alfalfa	0–6	*L. plantarum*	45	3
Wang et al. (2022)	34	2	Corn	0–5	*L. buchneri* and *L. plantarum*	60	4
Wang et al. (2021)	35	4	Paper mulberry	0–5	*L. buchneri* and *L. plantarum*	60	3
Guan et al. (2020b)	36	9	Corn	0–6	*L. salivarius, L. rhamnosus, L. salivarius*, and *L. rhamnosus*	60	3
Zhao S. et al. (2021)	37	2	Alfalfa	0–6		60	3
		185					

While creating the excel spreadsheet, datasets were divided into main categories which are main and branched cells. In the main cells, we included information on authors, year of publication, treatments, studies, doses, and substrates used as raw materials of the experience. Information on observed parameters was inserted in the branched cells of the created excel spreadsheet. The observed parameters include the chemical composition of silage material (DM, dry matter content; OM, organic matter; CP, crude protein; NDF, neutral detergent fiber; ADF, acid detergent fiber; WSC, water-soluble carbohydrates; EE, ether extract; ASH, the ash content in the fresh material; DM recovery, cellulose and hemicellulose; and ADL, acid detergent lignin), silage quality (pH value; LA, lactic acid; AA, acetic acid; PA, propionic acid; BA, butyric acid; AS, aerobic stability; LAB, lactic acid bacteria; AB, aerobic bacteria; yeasts, yeasts and molds, molds, and the starch content), silage microbiome, and information on sequencing. All the data of the targeted parameters were inserted into the created excel sheet to be ready for evaluation.

### 2.2. Statistical analysis

The articles were selected following the protocol of Preferred Reporting Items for Systematic Reviews and Meta-Analyses (PRISMA) (Moher et al., 2009; Mikolajewicz and Komarova, 2019), and data analysis were carried out using mixed model methodology as described by Abdelbagi et al. (2021) and Irawan et al. (2021). In this methodology, doses and LAB types included in the experiments were treated as a fixed factor, while the studies were treated as a random factor. Different values and means were accepted to be significant if the *p*-value is <0.05. As shown in Table 1, there were many types of substrates used for ensiling. In this study, to investigate the influence of the substrate, we calculated the interaction effects between the substrate and LAB types as well as doses of LAB, as presented in Tables 2, 3. The dataset presentation in figures was carried out using Microsoft excel 2013. Data of the silage microbiome were extracted from the figures using GetData digitizer version 2.26.0.20 software (http://getdata-graph-digitizer.com/). Before analyzing the relationships among response parameters and treatments, silage quality and silage microbiome were transformed into relative changes in treatment and control. The relationships between parameters and treatments were analyzed using hierarchical cluster analysis and were visualized using the heatmap.2 function from the *gplots* package in the R Console Version 4.2.1 (R Core Team, 2022).

**Table 2:** Interaction effects of lactic acid bacteria dose, types, and silage substrates on chemical composition and silage quality.

**Item**	**D**	**M**	**S**	D^*^M	D^*^S	M^*^S	D^*^M^*^S
DM	*	*	**	*	**	**	*
OM	NS	NS	**	NS	**	**	**
CP	NS	NS	**	NS	**	**	**
DM-recovery	NS	NS	NS	NS	NS	NS	NS
NDF	NS	NS	**	NS	**	**	**
ADF	NS	*	**	NS	**	**	**
EE	NS	NS	**	NS	**	**	**
ASH	NS	NS	-	NS	NS	NS	NS
ADL	NS	NS	NS	NS	NS	NS	NS
WSC	NS	NS	*	NS	**	*	*
Cellulose	*	*	**	*	**	**	**
Hemicellulose	NS	NS	**	*	*	*	*
pH	**	**	*	**	**	**	**
LA	**	**	**	*	**	**	**
AA	*	*	**	NS	*	**	**
PA	*	NS	**	*	NS	**	**
BA	*	*	**	NS	**	**	**
LA/AA	*	*	*	**	**	**	**
AS	*	*	-	NS	NS	NS	NS
NH3	**	**	*	**	**	**	**
LAB	*	*	**	NS	*	*	*
Coliform	*	*	**	*	**	**	**
Yeast and Mold	NS	NS	*	*	*	*	NS
Yeasts	*	*	NS	*	NS	*	NS
Molds	NS	NS	*	NS	NS	NS	NS
AB	NS	NS	NS	NS	NS	NS	NS
*L. plantarum*	NS	NS	*	NS	*	*	*
*L. buchneri*	NS	NS	NS	NS	NS	NS	NS
*Weissella*	NS	NS	NS	NS	NS	NS	NS
Weight lost	NS	NS	**	NS	**	**	**
Ethanol	NS	NS	NS	NS	NS	NS	NS

D, lactic acid bacteria dose; M, lactic acid bacteria types; S, silage substrate; D^*^M, the interaction effect among the lactic acid bacterial dose and the lactic acid type; D^*^S, the interaction effect among the lactic acid bacterial dose and the substrate; M^*^S, the interaction effect among the lactic acid type and the substrate; M^*^D, the interaction effect among the lactic acid bacteria type and the substrate; D^*^M^*^S, the interaction effect among the lactic acid bacteria dose, lactic acid bacterial type, and the substrate; DM, dry matter; OM, organic matter; CP, crude protein; NDF, neutral detergent fiber; ADF, acid detergent fiber; EE, ether extract; WSC, water-soluble carbohydrates; ADL, acid detergent lignin; LA, lactic acid; EB, aerobic bacteria; AA, acetic acid; PA, propionic acid; BA, butyric acid; LA/AA, the ratio of lactic to acetic acid; AS, aerobic stability; AB, aerobic bacteria; NS, not significant; ^*^when the p-value is less between 0.05 and 0.001; ^**^when the p-value is < 0.001; -, when the value is not detected.

**Table 3:** Interaction effects of the lactic acid dose, microbe type, and substrate on the bacterial diversity of the silage.

**Item**	**D**	**M**	**S**	D^*^M	D^*^S	M^*^S	D^*^M^*^S
Sequences	NS	NS	*	NS	NS	*	*
Shannon	NS	*	**	NS	*	*	NS
Simpson	NS	NS	**	NS	**	**	**
Chao	NS	NS	**	**	*	*	**
OTUs	NS	NS	*	NS	NS	*	*
Good's coverage	NS	*	**	NS	*	**	**
ACE	NS	NS	NS	NS	NS	NS	NS
*Firmicutes (%)*	NS	NS	*	*	NS	*	*
*Proteobacteria (%)*	NS	NS	NS	NS	NS	NS	NS
*Bacteriodetes (%)*	NS	NS	NS	NS	NS	NS	NS
*Cyanobacteria (%)*	NS	NS	*	NS	NS	NS	NS
*Lactobacillus (%)*	NS	*	*	*	*	*	*
*Leuconostoc (%)*	NS	NS	*	NS	NS	NS	NS
*Lactococcus (%)*	NS	NS	NS	NS	NS	*	*
*Pediococcus (%)*	NS	NS	NS	NS	*	*	*
*Acinetobacter (%)*	NS	NS	NS	NS	NS	NS	NS
*Bacillus (%)*	*	*	NS	NS	NS	NS	NS
*Weissella (%)*	NS	NS	NS	NS	*	NS	*
*Pseudomonas (%)*	NS	NS	*	*	**	**	**
*Clostridium (%)*	NS	NS	NS	NS	NS	NS	NS
*Enterobacter (%)*	NS	NS	NS	NS	NS	-	-
*Enterococcus (%)*	NS	*	NS	**	**	*	**
*Klebsiella (%)*	NS	NS	NS	**	*	*	**
*L. plantarum (%)*	NS	NS	NS	NS	*	*	*
*L.brevis*	NS	NS	NS	NS	NS	NS	NS
*L. buchneri (%)*	NS	NS	NS	NS	NS	NS	NS
*Acetobacter* spp. *(%)*	NS	NS	**	*	*	*	*
*Sphingobacterium* spp. *(%)*	NS	NS	NS	NS	*	NS	NS

NS, not significant; ^*^when the P-value is < (0.05); ^**^when the P-value is < (0.001). ACE, abundance-based coverage estimator; OTUs, operational taxonomic units. D, lactic acid bacteria dose; M, lactic acid bacteria types; S, silage substrate.

## 3. Results

The studies used for structuring the meta-analysis database are shown in Table 1. Descriptive statistic of the chemical composition of raw materials before the ensiling process is presented in Table 4. Effects of LAB dose on silage microbiome and silage quality are presented in Tables 2, 5, respectively. Effects of LAB types on silage microbiome and silage quality are shown in Tables 6, 7, respectively. Interaction effects among LAB doses, LAB types, and the raw material on silage microbiome and silage quality are given in Tables 2, 3, respectively. The differences in substrates in the silage affected the chemical composition and quality of silages (Table 2) as well as the value of Shannon, Simpson, OTUs, and some bacteria (Table 3).

**Table 4:** Descriptive statistics of the substrate prior to the ensiling process.

**Item**	**Unit**	**Mean±Stdev**	**Min**	**Max**
DM	g/kg	303.4 ± 92.3	124.8	589.3
OM	g/kg DM	910.15 ± 28.72	850.1	948
CP	g/kg DM	136.67 ±81.65	30.2	299
NDF	g/kg DM	486.04 ±128.56	317.2	950
ADF	g/kg DM	292.23 ±118.60	106.0	666.1
EE	g/kg DM	36.14 ±18.79	9.9	64.5
WSC	g/kg DM	64.30 ±35.80	7.8	177.7
ADL	g/kg DM	59.22 ±8.33	45.2	65.6
Hemicellulose	g/kg DM	172.28 ±46.81	139.8	293.9
Xylose	g/kg DM	0.80 ±0.14	0.7	0.9
pH		5.93 ±0.59	4.2	7.0
LA	g/kg DM	7.61 ±0.83	6.7	8.9
AA	g/kg DM	5.26 ±2.23	2.1	9.0
BA	g/kg DM	3.48 ±1.89	1.4	5.1
LAB	Log CFU/g	3.61 ±2.25	1.0	8.6
AB	Log CFU/g	6.62 ±1.86	4.3	9.6
EB	Log CFU/g	7.32 ±1.00	6.3	8.3
CFB	Log CFU/g	4.04 ±2.25	1.0	7.3
Yeast	Log CFU/g	3.62 ±2.07	1.0	6.9
Molds	Log CFU/g	2.58 ± 1.66	1.0	6.8

DM, dry matter; OM, organic matter; CP, crude protein; NDF, neutral detergent fiber; ADF, acid detergent fiber; EE, ether extract; WSC, water-soluble carbohydrates; ADL, acid detergent lignin; LAB, lactic acid; LA, lactic acid; AA, acetic acid; BA, butyric acid, AB, aerobic bacteria; EB, enterobacteria; CFB, coliform bacteria; Min, minimum; Max, maximum; Stdev, standard deviation; CFU, colony-forming unit.

**Table 5:** Effects of lactic acid bacteria on chemical composition and fermentation characteristics post-ensiling process.

**Item**	**n**	**Unit**	**Model**	**Intercept**	**SE. intercept**	**Slope**	**SE. slope**	**Trend**	**AIC**	***p*** **-value**
DM	214	g/kg	L	307.89	10.4462	0.7700	0.2531	+	2,024.6	0.0026
OM	28	g/kg DM	L	910.44	8.3158	0.9826	1.5631	+	244.8	0.6100
CP	162	g/kg DM	L	134.29	8.3296	0.2657	0.3152	+	1516.7	0.4014
DM-Recovery	22	%	L	92.8052	1.1570	0.07666	0.09767	+	112.9	0.4507
NDF	163	g/kg DM	L	526.86	17.1251	−0.6357	0.8583	-	1,813.3	0.4607
ADF	163	g/kg DM	L	341.41	12.4237	−0.8419	0.4787	-	1,659.4	0.0818
EE	15	g/kg DM	L	21.4983	6.6532	0.1491	0.1580	+	87.2	0.3730
Ash	12	g/kg DM	L	3.3617	0.7208	−0.09370	0.06927	-	49.2	0.2341
ADL	20	g/kg DM	L	67.6235	3.9693	−1.0327	0.8253	-	251.8	0.2329
WSC	115	g/kg DM	L	30.2105	3.6588	−0.2001	0.2733	-	910.8	0.4665
Cellulose	14	g/kg DM	L	322.68	17.2706	−1.2582	0.1498	-	113.3	0.0004
Hemicellulose	48	g/kg DM	L	175.54	17.1905	−0.3415	0.4576	-	432.5	0.4612
pH	261		L	4.9539	0.08640	−0.05184	0.006163	-	397.4	< 0.0001
LA	239	g/kg DM	L	28.1404	2.0670	0.8982	0.1443	+	1,865.7	< 0.0001
AA	214	g/kg DM	L	13.2626	1.3373	0.3696	0.1464	+	1,545.9	0.0128
PA	154	g/kg DM	L	6.4569	1.6825	0.08592	0.03497	+	843.6	0.0159
BA	115	g/kg DM	L	4.1009	0.8249	−0.2129	0.09930	-	693.4	0.0360
LA/AA	73	g/kg DM	L	1.7011	0.4591	0.3254	0.08262	+	344.3	0.0003
AS	18	h	L	31.1285	5.1683	−0.1993	0.8724	+	119.3	0.8224
NH3	199	g/kg DM	L	58.1511	5.5604	−2.0707	0.3541	-	1833.4	< 0.0001
LAB	130	Log CFU/g	L	6.6271	0.2397	0.07751	0.03495	+	480.9	0.0297
Coliform	83	Log CFU/g	L	3.6174	0.3505	−0.1375	0.03529	-	295.6	0.0003
Yeast and Mold	38	Log CFU/g	L	2.8501	0.3076	−0.03684	0.03815	-	109.5	0.3443
Yeasts	74	Log CFU/g	L	3.6483	0.3019	−0.1274	0.04629	-	260.1	0.0088
Molds	44	Log CFU/g	L	2.1130	0.2901	0.04138	0.04403	+	128.5	0.3571
AB	36	Log CFU/g	L	3.4561	0.3979	−0.05959	0.03976	-	119.9	0.1512
*L. plantarum*	13	Log CFU/g	L	5.5294	2.3087	−0.2314	0.1616	-	65.3	0.2020
*L. buchneri*	14	Log CFU/g	L	1.0364	0.3712	0.03532	0.07715	+	39.0	0.6592
*Weissella*	13	Log CFU/g	L	3.2319	1.0542	−0.1059	0.2156	-	60.9	0.6407
Weight lost	53	g/kg DM	L	18.3260	7.4001	0.08222	0.1699	+	418.0	0.7928
Ethanol	16	g/kg DM	L	10.0745	5.0605	−0.01309	0.1583	-	219.9	0.0402

DM, dry matter; OM, organic matter; CP, crude protein; NDF, neutral detergent fiber; ADF, acid detergent fiber; EE, ether extract; WSC, water-soluble carbohydrates; ADL, acid detergent lignin; LA, lactic acid; LAB, lactic acid; AA, acetic acid; PA, propionic acid; BA, butyric acid, LA: AA, the ratio of lactic to acetic acid; AS, aerobic stability; AB, aerobic bacteria; AIC, Akaike information criterion; L, linear pattern.

**Table 6:** Effects of lactic acid bacterial types on the chemical composition and silage quality.

**Item**	**No**	**Unit**	**CTRL**	**LAB**	**COM**	***p*** **-value**
DM	214	g/kg	306.82	313.89	312.34	0.0007
OM	28	g/kg DM	908.71	907.77	926.74	0.4220
CP	162	g/kg DM	134.18	137.28	133.63	0.3240
DM-Recovery	22	%	92.4699	93.1813	96.1000	0.4874
NDF	163	g/kg DM	527.84	517.81	527.95	0.2612
ADF	165	g/kg DM	336.71	327.45	336.48	0.0272
EE	15	g/kg DM	21.5593	22.1059	22.9382	0.6347
ASH	12	g/kg DM	3.3617	2.5183	-	0.2341
ADL	30	g/kg DM	67.7421	58.1200	60.7100	0.4922
WSC	115	g/kg DM	30.3547	27.6166	30.9593	0.1897
Cellulose	14	g/kg DM	323.67	309.51	311.86	0.0003
Hemicellulose	48	g/kg DM	176.27	176.00	168.74	0.0750
Ph	261		4.9782	4.6286	4.5727	< 0.0001
LA	239	g/kg DM	27.9236	33.8525	34.5152	< 0.0001
AA	214	g/kg DM	13.1869	15.9695	14.3715	0.0234
PA	154	g/kg DM	6.4944	7.0160	6.8894	0.1078
BA	115	g/kg DM	4.2656	2.3239	2.8861	0.0381
LA/AA	73	g/kg DM	2.0932	2.8612	4.8754	0.0071
AS	18	H	31.1286	29.9330	-	0.8224
NH3	199	g/kg DM	58.3157	45.3725	48.5366	< 0.0001
LAB	130	Log CFU/g	6.5686	6.9669	7.4774	0.0217
Coliform	83	Log CFU/g	3.6038	2.8117	3.0073	0.0022
Yeast and Mold	38	Log CFU/g	2.8853	2.5891	2.8127	0.5654
Yeasts	74	Log CFU/g	3.6439	2.8857	3.0257	0.0364
Molds	44	Log CFU/g	2.1123	2.3131	2.4001	0.6940
AB	36	Log CFU/g	3.4588	3.1668	2.9645	0.3413
*L. plantarum*	13	Log CFU/g	5.5764	4.3714	3.0164	0.2207
*L. buchneri*	14	Log CFU/g	1.0364	1.4203	0.9045	0.6371
*Weissella*	13	Log CFU/g	3.1810	2.7835	2.8088	0.9604
Weight lost	53	g/kg DM	18.4898	18.7899	17.5138	0.9841
Ethanol	16	g/kg DM	10.0965	10.0407	9.9556	0.9903

Means of different lactic acid bacteria inoculants. CTRL, control treatment; LAB, lactic acid bacteria; COM, the combination of lactic acid bacteria; DM, dry matter; OM, organic matter; CP, crude protein; NDF, neutral detergent fiber; ADF, acid detergent fiber; EE, ether extract; WSC, water-soluble carbohydrates; ADL, acid detergent lignin; LA, lactic acid; EB, aerobic bacteria; AA, acetic acid; PA, propionic acid; BA, butyric acid; LA:AA, the ratio of lactic to acetic acid; AS, aerobic stability; AB, aerobic bacteria; L, linear pattern; quadratic pattern; -, when the value is not detected.

**Table 7:** Effects of lactic acid bacterial addition on the relative abundance and bacterial communities' post-ensiling process (%).

**Item**	***n***	**Model**	**Intercept**	**SE. intercept**	**Slope**	**SE. slope**	**Trend**	**AIC**	***p*** **-value**
Sequences	74	L	51,975	2,839.1371	−219.61	244.22	-	1,971.2	0.3731
Shannon	125	L	2.7038	0.2650	−0.04878	0.02367	-	392.5	0.0426
Simpson	74	L	0.7793	0.1598	−0.00061	0.004586	-	6.3	0.8954
Chao	103	L	319.06	73.3632	−1.5508	3.9776	-	1,388.6	0.6979
OTUs	70	L	343.54	82.5750	−5.3052	6.1125	-	933.2	0.3903
Good's coverage	94	L	0.9895	0.001563	0.000437	0.000240	+	−612.4	0.0747
ACE	25	L	209.10	43.2387	−5.8275	8.1224	-	308.8	0.4868
*Firmicutes (%)*	32	L	60.7197	8.4082	1.6578	1.2276	+	296.3	0.1956
*Proteobacteria (%)*	28	L	26.7514	6.3435	−1.5304	1.0741	-	243.8	0.1761
*Bacteriodetes (%)*	21	L	3.4053	1.2526	0.07538	0.3928	-	102.1	0.8533
*Cyanobacteria (%)*	10	L	4.0528	0.9501	0.1673	0.1201	+	42.9	0.2360
*Lactobacillus (%)*	93	L	48.7638	5.1754	1.0194	0.5747	+	842.6	0.0817
*Leuconostoc (%)*	17	L	8.9116	6.3273	0.005718	1.5091	+	138.6	0.9971
*Lactococcus (%)*	40	L	20.8089	7.5305	0.6518	0.8054	+	391.3	0.4266
*Pediococcus (%)*	35	L	14.8045	3.4450	−0.1374	0.3092	-	260.6	0.6632
*Weissella (%)*	48	L	9.3509	3.7827	−0.2075	0.2766	-	363.2	0.4610
*Bacillus (%)*	10	L	1.2862	0.1923	0.04831	0.01292	+	17.3	0.0334
*Acinetobacter (%)*	19	L	4.4357	1.5185	−0.1224	0.3109	-	115.6	0.7075
*Pseudomonas (%)*	30	L	11.1301	2.9112	−0.8362	0.4757	-	213.0	0.1814
*Clostridium (%)*	13	L	12.4335	5.0335	0.6272	1.0789	+	95.3	0.5862
*Enterobacter (%)*	50	L	6.2907	1.3678	−0.1222	0.2127	+	307.0	0.5704
*Enterococcus (%)*	35	L	9.5241	3.2499	−0.3176	0.3992	-	265.0	0.4360
*Klebsiella (%)*	15	L	1.7909	0.4576	−0.05210	0.07744	-	48.6	0.5227
*Acetobacter* spp. *(%)*	24	L	5.0109	1.7023	−0.01754	0.08943	-	128.3	0.8489
*L. plantarum (%)*	13	L	11.4470	10.4423	5.2478	2.5516	+	111.3	0.0854
*L. buchneri (%)*	12	L	22.6904	13.6594	2.6980	2.3817	+	104.5	0.3087
*L.brevis (%)*	11	L	5.2529	1.1582	−0.2039	0.3136	-	51.3	0.5510
*Sphingobacterium* spp. *(%)*	17	L	2.1133	1.1249	0.05406	0.2545	-	89.1	0.8388

L, linear pattern; n, number of the studies used; AIC, Akaike information criterion.

**Figure 1:**
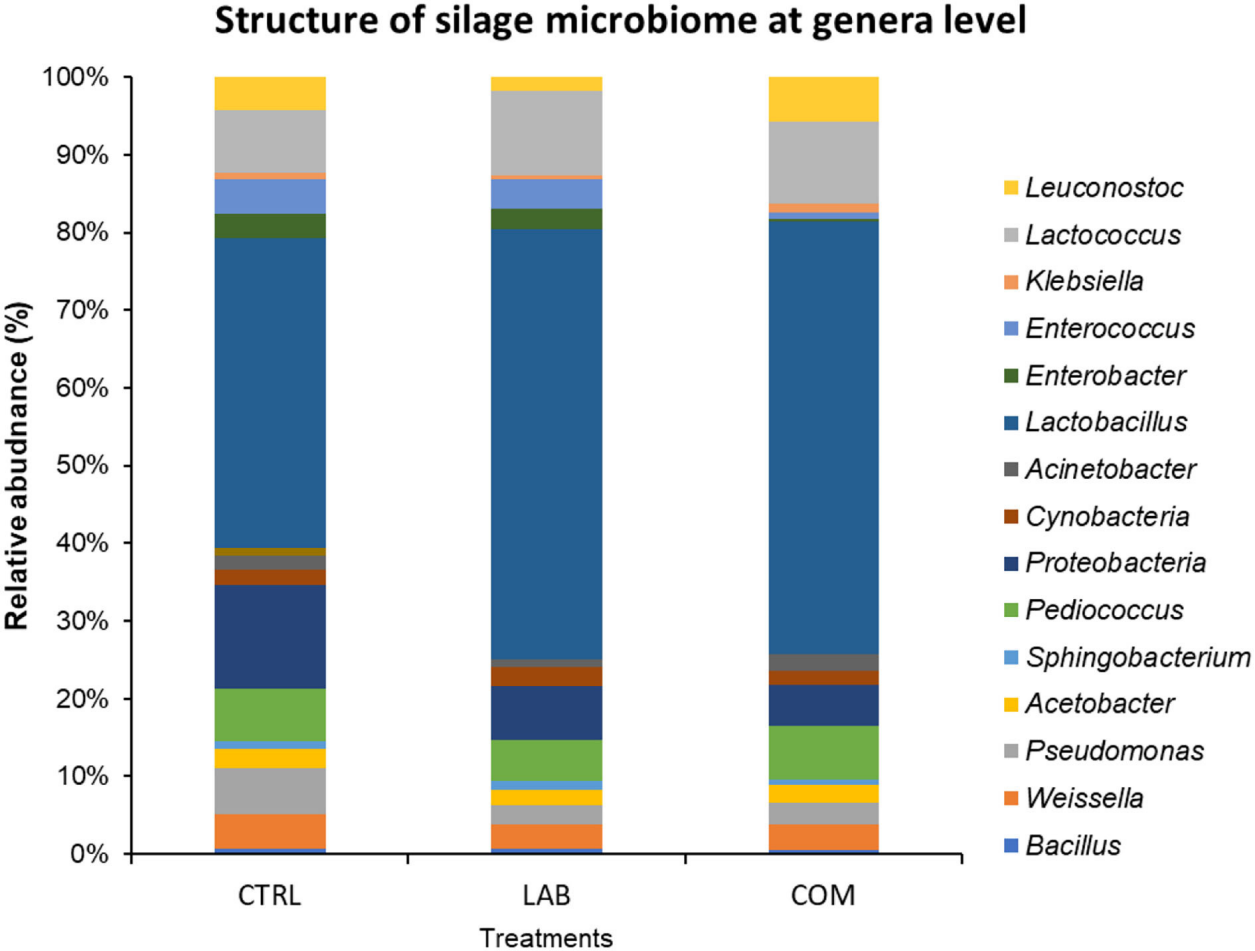
Comparison structure of silage microbiome at genera level between control and inoculant addition.

The patterns of the relationship between the characteristics of inoculum and the physicochemical properties of silage are visualized in Figure 1. The treatments showed to impact significantly on silage microbiome and silage quality. However, phylum *Firmicutes* and the genera of *Pediococcus, Proteobacteria, Pseudomonas*, and *Weissella* dominated the post-ensiling process. Either LAB or combined LAB group in the silage increased the abundance of LAB, especially *Lactobacillus*; however, *Proteobacteria* and non-lactic acid-producing bacteria become lower than those in control (Figure 1). Domination of LAB in inoculated silage implied diversity index decreased (Table 8). Moreover, both *L. plantarum* and *L. buchneri* were increased, and subsequently, *Weissella, Pseudomonas, Proteobacteria*, the pH, and the ammonia nitrogen decreased significantly due to the addition of LAB in the silage (*p* < 0.05) (Table 8). However, LA, AA, the ratio of LA to AA, and LAB counts enhanced significantly due to the inclusion of LAB inoculants (*p* < 0.05) (Table 6).

**Table 8:** Effects of lactic acid bacterial types on the relative abundance bacterial communities of silage post-ensiling process (%).

**Item**	***n***	**CTRL**	**LAB**	**COM**	***p*** **-value**
Sequences	74	51,876	51,121	50,630	0.7133
Shannon	125	2.7017	2.4581	2.3488	0.1461
Simpson	74	0.779	0.7779	0.7605	0.9432
Chao	103	317.95	309.83	312.52	0.9597
OTUs	70	342.76	320.47	310.88	0.7157
Good's coverage	94	0.9891	0.9937	0.9898	0.0266
ACE	25	202.38	104.26	252.33	0.2157
*Firmicutes (%)*	32	60.0324	64.3572	80.2195	0.2657
*Proteobacteria (%)*	28	28.8811	17.5945	12.4154	0.2176
*Bacteriodetes (%)*	18	3.5162	5.5023	1.6937	0.7953
*Cyanobacteria (%)*	10	4.1000	6.0916	3.9550	0.2913
*Lactobacillus (%)*	93	46.1536	56.2316	60.6151	0.0166
*Leuconostoc (%)*	17	8.9819	4.2917	13.5975	0.7928
*Lactococcus (%)*	40	17.5354	27.3373	24.5841	0.3958
*Pediococcus (%)*	35	14.5268	13.1416	16.4107	0.6321
*Bacillus (%)*	10	1.2846	1.8006	1.3243	0.0468
*Weissella (%)*	48	9.8673	7.8929	7.7889	0.6274
*Pseudomonas (%)*	30	12.7930	6.1339	6.4381	0.1836
*Acetobacter* spp. *(%)*	24	5.1354	4.7769	5.3908	0.9095
*Acinetobacter (%)*	19	4.2207	2.5250	5.1929	0.7532
*Clostridium (%)*	13	10.8292	-	7.4685	0.4779
*Enterobacter (%)*	50	6.8024	6.6190	1.0671	0.0206
*Enterococcus (%)*	35	9.7216	9.0838	1.9079	0.3386
*Klebsiella (%)*	15	1.7982	1.3445	2.5503	0.4412
*L. plantarum (%)*	13	11.4103	39.6147	24.9581	0.2129
*L. buchneri (%)*	12	22.8550	38.5621	38.6874	0.6080
*L. brevis*	11	5.2901	3.6577	6.0895	0.6018
*Sphingobacterium* spp. *(%)*	17	2.1919	2.7195	1.4565	0.9304

Means of different lactic acid bacteria inoculants; CTRL, control treatment; LAB, lactic acid bacteria; COM, the combination of lactic acid bacteria; n, number of the studies; ACE, abundance-based coverage estimator; OTUs, operational taxonomic units.

Among LAB types, the lowest abundance of *Pseudomonas* was scored when treated with the LAB group, while the lowest abundance of *Weissella* and *Proteobacteria* was due to the addition of the combined LAB group. There was no significant effect of treatments on WSC, hemicellulose, and aerobic stability. Treatment with the combined LAB group (COM) has resulted in the lowest pH value, the highest concentration of LA, the highest ratio of LA to AA, and the highest LAB count in silage materials as compared with the LAB treatment. In contrast, the lowest concentration of NH_3_-N and the highest concentration of LAB in silage materials were obtained due to the inclusion of the LAB group.

**Figure 2:**
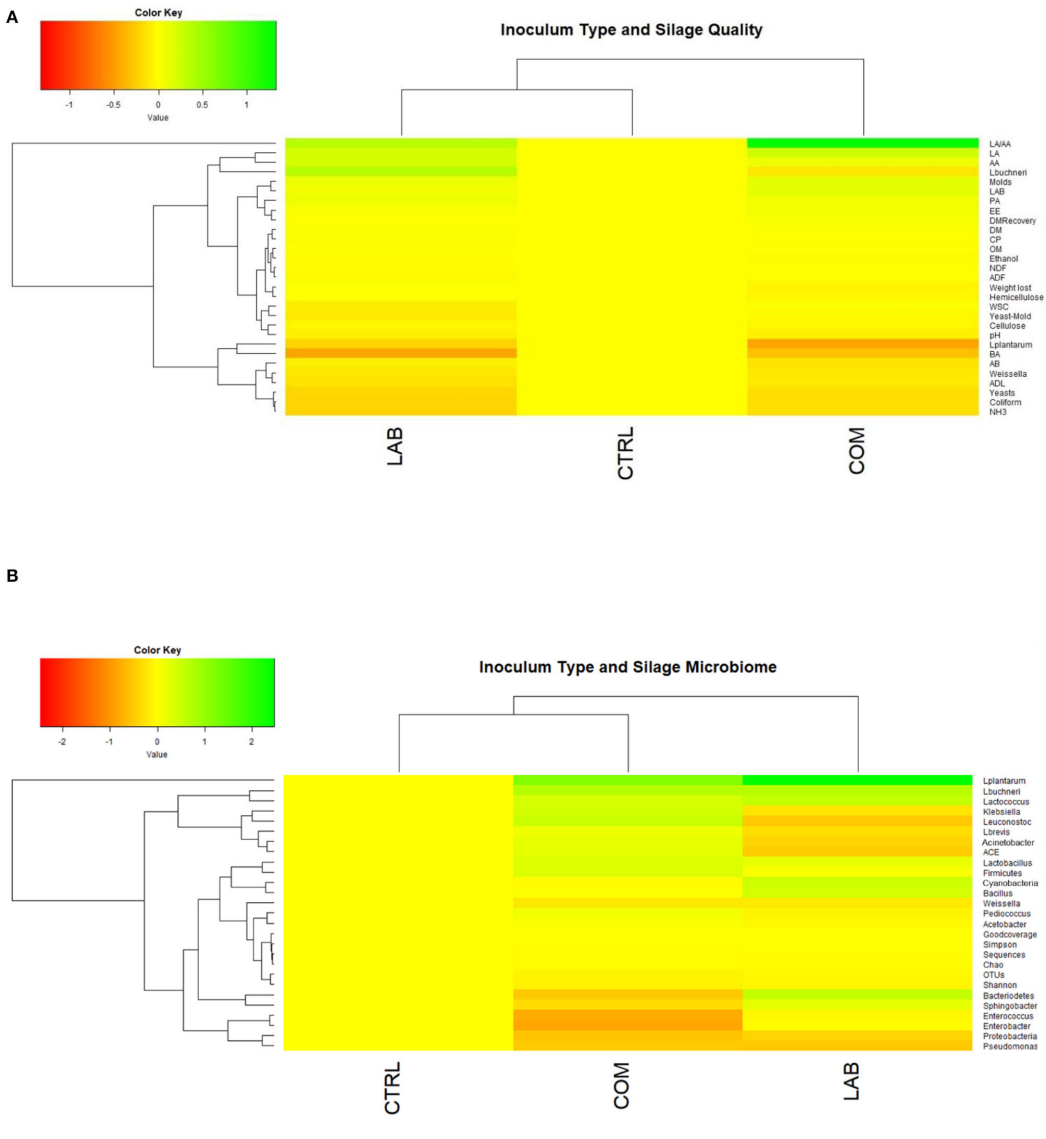
Dendro-heatmap visualized the relationship patterns between inoculum types addition with silage quality **(A)** and between inoculum types addition with silage microbiome **(B)**. Colors key indicates the treatment effect from declining (red) to increasing (green) compared to the control. CTRL, control treatment without lactic acid bacterial inoculant; LAB, lactic acid bacteria addition; COM, the addition combining different lactic acid bacterial types. DM, dry matter; OM, organic matter; CP, crude protein; NDF, neutral detergent fiber; ADF, acid detergent fiber; EE, ether extract; WSC, water-soluble carbohydrates; ADL, acid detergent lignin; LA, lactic acid; AA, acetic acid; PA, propionic acid; BA, butyric acid; LA/AA, ratio of lactic to acetic acid; AB, aerobic bacteria; ACE, abundance-based coverage estimator; OTUs, operational taxonomic units.

Patterns of the relative change in silage quality and microbiome parameters for each treatment are visualized in Figure 2. The increased organic acid (LA and AA) and the decreased acetic acid in the treated silage were categorized in the contrast cluster (Figure 2A), while dynamic changes in non-lactic acid bacteria in the treated silage were categorized in the same cluster (Figure 2B).

## 4. Discussion

The chemical composition of raw materials before the ensiling process is shown in Table 4. The mean values of DM, CP, and WSC were 303.4, 136, and 64 g/kg FW, respectively, which were in the same range as that in the previous report of Wang X. et al. (2022). It is stated that several factors could influence forage content, including fertilization, the season of harvesting, irrigation, and sowing density (Mu et al., 2020).

Based on the results of this study, it can be seen that the addition of LAB has decreased significantly the richness of the Shannon index and influenced the microbiome of silage materials (*p*<*0.05*). As shown in Tables 7, 8, *Firmicutes, Pediococcus, Proteobacteria, Pseudomonas*, and *Weissella* are the greatest composer of silage microbiome of both control and LAB treatments post-ensiling process. When LAB inoculants were added, the relative abundances of *Pseudomonas, Proteobacteria*, and *Weissella* were reduced by ~50, 38, and 20%, respectively. The reduction of the Shannon index is associated with the rapid decline of pH value, resulting in inhibiting undesirable microorganisms. The result was in line with Ridwan et al. (2015) and Mu et al. (2022), who reported a significant decrease in the Shannon index due to the addition of LAB inoculants in the silage. In contrast, the reduction of *Weissella* spp. is attributed due to the lack of compatibility between additives and raw materials (Mu et al., 2022).

It is reported that *Weissella* spp. commonly present in the fresh forage or silage, initiating lactate fermentation in silage and creating an appropriate environment for developing the *Lactobacilli* (Mu et al., 2022). Similarly, *Weissella, Pediococcus*, and *Lactococcus* are the predominant bacteria; thereafter these bacteria would be shifted gradually by *Lactobacilli* due to the greater decline of pH value (Mu et al., 2020). In contrast, the abundance of *Firmicutes* mass was increased by 7% and 33% in LAB treatment and the combination of different types of LAB treatment. This is perhaps because *Firmicutes* is a great acid-producing hydrolytic phylum that can grow quickly at low pH conditions (Chen et al., 2020). The same result was observed by Zi et al. (2021).

Moreover, LAB inoculants have decreased the relative abundance of *Enterococcus*. The result was consistent with Zhang et al. (2021). Compared with LAB treatments, the relative abundance of *Cyanobacteria, Acinetobacter*, and *Enterobacter* in the control silage was approximately about 4.1, 4.2, and 6.8, respectively. The dominance of *Cyanobacteria* in the control silage indicates the worth condition of the silage (He et al., 2020). Therefore, the addition of combined species of LAB could be used in the future as an effective method for improving silage conditions, resulting in improving silage environments and improving bacterial dynamics. *Cyanobacteria* are known as a photosynthesizing phylum of bacteria found in diverse environments with prolific growth and a wide variety of generated products. Furthermore, the addition of LAB inoculants has numerically reduced the abundance of *Enterobacter*, while the lowest abundance was scored due to the addition of the combination of different LAB species. Therefore, this indicates that LAB inoculants could prevent silage from spoilage. It is reported that *Enterobacter* converts LA to AA and other organic acids (Zi et al., 2021). *Acetobacter* spp. contribute to producing anaerobic conditions. The genus *Enterobacter* is known to cause silage spoilage (Jaipolsaen et al., 2022).

As shown in Tables 5, 7, the addition of LAB is effective in improving silage preservation by increasing the abundance of *Lactobacillus, L. buchneri*, and *L. plantarum*. It is known that some *Lactobacillus* is a homofermentative LA-producing bacteria, with a key role in inhibiting microbial activity by rapid acidification in ensiling proses (Mu et al., 2020). Therefore, we observe a numerical reduction of *Clostridium* spp., coliform, and yeasts due to the addition of LAB inoculants in the silage (Table 8). The addition of LAB has been proven to improve silage quality significantly by increasing LA, AA, the ratio of LA to AA, and the LAB count in the silage and decreasing pH value, NH_3_-N, and cellulose. The reduction of pH value is associated with inhibiting the undesirable microorganisms, including coliform, and the yeasts and some LAB, such as streptococci, in silage due to their low tolerance to the lower pH condition (He et al., 2019). The result was in line with Guo et al. (2020).

Indeed, the decrease of NH_3_-N is due to the effects of *Pseudomonas* and *Proteobacteria. Pseudomonas* plays a crucial role in degrading organic materials, while *Proteobacteria* are essential in degrading the CP, resulting in lower NH_3_-N (Mu et al., 2022). *Enterobacter* spp. can ferment amino acids and produce NH_3_-N. So, all these species function to reduce the concentration of the NH_3_-N of silage materials. In contrast, the high concentration of NH_3_-N in silage is a good indicator of the excessive breakdown of protein in silage. The NH_3_-N mass which is above 40 g/kg indicates a good quality of silage materials (Mu et al., 2020). Moreover, Li P. et al. (2018) and Guan et al. (2020a) reported a significant decrease in the number of both the coliform and the yeasts as affected by the addition of the LAB.

In contrast, we did not observe significant effects of LAB addition on WSC. WSC is considered a limiting factor for LA production. It is known that LAB can metabolize the WSC into various organic acids, leading to produce more LA which significantly improves the conditions for the ensiling process (Chen et al., 2021). In addition, this response might also be due to the differences in substrates used in the silage. In this meta-analysis study, the substrates affected the WSC concentration. However, the present study is concerned with the influence of LAB and its mixtures on silage quality and bacterial diversity. Therefore, further studies are needed to diverge certain substrates used in the silage.

It is suggested that many factors could influence the microbial diversity of silage, including raw materials, environmental temperature, and type of inoculant. For example, raw materials were found to significantly influence bacterial abundance at the genus and phylum level in different ways (Du et al., 2021). In addition, *Pantoea agglomerans, Pseudomonas* spp., *Pseudomonas koreensis, Serratia liquefaciens*, and *Pseudomonas coleopterorum* were suggested to be the most dominant species of silage made from corn (Du et al., 2021), while *Acinetobacter* spp*., P. agglomerans, Enterobacter* spp., *Streptomyces alboniger*, and *L. plantarum* were the most undetectable species of silage material post-ensiling process. The addition of *L. plantarum* in Sainfoin silage has been shown to promote the growth rate of *L. acetotolerans, L. buchneri, L. plantarum, L. pentosus*, and *Clostridium tyrobutiricum* (Xu et al., 2020). Furthermore, *Lactobacillus* spp. and *Bacillus* sp. were the most common spoilage organisms in silage of welted rice straw that is treated by *L. plantarum* (Wang et al., 2016).

The addition of *L. plantarum* also has been shown to reduce microbial diversity as indicated by the reduction of the Shannon index. Previous research conducted a study on the effects of different regions or different LAB types on the whole-plant maize silage. The authors found that *Weissella* was the dominant epiphytic bacteria of raw materials Ziyun and Weinning, while *Lactobacillus* was prevalent in Guanling (Huang et al., 2021). In contrast, the effects of environmental temperature on bacterial diversity and the fermentation process were previously studied by Wang et al. (2019). The study concluded that *Pseudomonas, Exiguobacterium*, and *Acinetobacter* were more abundant in silages stored at 30°C than 15°C. In addition, Zhang (2018) observed that the inclusion of LAB improved the fermentation quality of alfalfa silage stored at 20°C and 30°C, while ensiling of alfalfa at 40°C is difficult because of *Garciella*. Since both *Pseudomonades* and *Exiguobacterium* are undesirable strains, the relatively lower temperature is an effective method for preserving silage materials. This means that the low temperature, i.e., between 15°C and 30°C, is another technology that could be used effectively to inhibit the undesirable microbes in silage. Therefore, this indicates that many factors could influence the bacterial communities of silage after the ensiling process, including raw material, LAB inoculants, and environmental temperature. The addition of LAB inoculants has been shown to increase the abundance of *Lactobacillus* and increase the abundance of *L. plantarum* and *L. buchneri* (Figures 1, 2B). This has resulted in enhancing silage quality by increasing LAB count and subsequently LA, AA, and the ratio of LA to AA. The increase of LAB has resulted in a significant reduction of *Coliform*, yeasts, and NH_3_-N concentration of silage materials. This was consistent with Mu et al. (2020), Mu et al. (2022), and Xiong et al. (2022).

According to Mu et al. (2022), the addition of LAB has resulted in a positive correlation with *W. cibaria, Erwinia* sp*., Ewingella americana, Paenibacillus* sp., and *L. acetotolerans*. On the contrary, *L. buchneri, L. plantarum, L. paralimentarius, L. buchneri*, and *L. nodensis* were negatively correlated with *Erwinia* sp*., Ewingella americana*, and *L. acetotolerans* that can survive under lower pH conditions, while *Enterobacteriaceae* can survive and compete with LAB to utilize the WSC as a primary energy source (Pereira et al., 2007). It is reported that *Enterobacteriaceae* degrade LA into acetate, succinate, and some endotoxins (Zhao S. et al., 2021; Wang W. et al., 2022). Wang et al. (2022) found a positive correlation between *Enterobacteriaceae*, pH, and NH_3_-N, while LA and WSC were negatively correlated. *Enterobacter* and LAB compete with each other for energy sources of WSC, but which factor causes the dominance of LAB is still unknown yet. It is probably due to the facultative anaerobic nature of *Enterobacteriaceae*. Anaerobic conditions provoke *Enterobacteriaceae* to use WSC as a fermentative substrate, while oxygen exposure conditions of the respiratory process become dominant (Sarkar and Mohan, 2020). Molecular approaches such as NGS are one of the tools that have provided many insights into investigating microbiome diversity in silage fermentation. Indicators of the alpha diversity index, including Chao1 and Shannon, were used to determine the relative abundance and diversity indices of bacterial communities in the silages (Wang X. et al., 2022). Due to the lack of information on the correlation between bacterial communities of silage and the physiochemical properties of silage, this study has limitations.

## 5. Conclusion

The LAB inoculants are effective means of altering the bacterial community of silage, i.e., enhancing the dominance of *Lactobacillus* spp. and decreasing the diversity index of silage microbes. Phylum *Firmicutes* and genera *Pediococcus, Proteobacteria, Pseudomonas*, and *Weissella* are the most dominant bacteria of silage materials. Moreover, LAB inoculants are an effective method in elevating silage fermentation quality by increasing the relative abundance of *L. plantarum* and *L. buchneri*, LA, AA, the ratio of LA to AA, and LAB counts, and decreasing the pH and NH_3_-N values of silage materials. Therefore, our results suggest that LAB inoculants are the best recommendation for improving sustainable feed preservation and silage quality by altering the bacterial communities and enhancing favorable fermentation products during ensiling.

## Data availability statement

The original contributions presented in the study are included in the article/supplementary material, further inquiries can be directed to the corresponding author.

## Author contributions

RR: supervision, reference collection, investigation, rechecking the metadata, manuscript correction, and correlation analysis. MA: data collection, data input, investigation, correlation analysis, statistical analysis, and writing the original draft. AS: figure visualization, reference collection, rechecking the metadata, and manuscript correction. RF, WA, AF, MS, R, and KS: reference collection, investigation, rechecking the metadata, correlation analysis, and manuscript correction. AJ: rechecking the metadata, correlation analysis, and manuscript correction. YW: supervision, investigation, rechecking the metadata, and writing and reviewing the manuscript. All authors contributed to the article and approved the submitted version.
